# Clinical Review, and Hospital Reports

**Published:** 1838-07-01

**Authors:** 


### Clinical Review.

### Guy's Hospital.

### ON ABDOMINAL TUMORS.

Guy's Hospital Reports. Edited by *George Barlow*, M.A. &c. &c. and *James P. Babington*, M.A. &c. &c. Nos. V. and VI.

1. Observations on Abdominal Tumors and Intumescence: Illustrated by some Cases of Acephalocyst Hydatids. By *Dr. Bright.*
2. Observations on Abdominal Tumors and Intumescence: Illustrated by Cases of Ovarian Disease. By the same.

All practical physicians and surgeons are aware of the difficulty which usually hangs upon the discrimination of abdominal tumors. Any attempt at the diminution of that difficulty is meritorious—any degree of success attending it must be a subject of congratulation.

Dr. Bright informs us that it is his intention to devote several papers to the consideration of abdominal tumors and intumescence. He has actually offered two, and we trust will present, on future occasions, more.

The topics, he says, which naturally present themselves, admit of being arranged under the following heads:—1. The Integuments; 2. The Peritoneum; 3. The Stomach; 4. The Intestines; 5. The Liver; 6. The Spleen; 7. The Pancreas; 8. The Mesenteric glands; 9. The Kidneys; 10. The Bladder; 11. The Uterus; 12. The Ovaries; 13. Extra-Uterine Bodies; 14. Aneurism. But although the particular subjects for consideration are enumerated in this order, Dr. Bright disclaims a methodical pursuit of it.

Symptoms, we all know, are the language of disease. The special and the general phenomena which form the symptoms of abdominal tumors, are the form and appearance presented to the eye; the form still further discovered by the touch; the resistance ascertained by pressure; the sounds elicited by percussion; and, in a few instances, the sounds perceptible to the ear, either alone or by the aid of the stethoscope: and besides these local and physical signs, we look to the general condition of the system, and of the various excretions, as rendering us most important assistance, and being frequently indispensable towards the formation of a tolerably correct diagnosis.

"In studying the local indications of disease," continues our excellent and able friend, "the first object is, of course, to learn and fix in our minds the exact normal position of each viscus, and the modifications of form, appearance, and resistance which the muscles of the parietes are capable of impressing upon the different parts of the abdomen. There is, however, one circumstance connected with the abdominal viscera, which must always throw a certain degree of doubt upon all physical diagnosis, as directed to this part of the body; and this is the diversity which sometimes takes place in the organs themselves, and that more particularly with regard to the colon; the arch and the sigmoid flexure of which not unfrequently form extensive convolutions, which render any inferences derivable from its natural position somewhat doubtful. The liver, likewise, occasionally deviates from its ordinary situation and form; and, in rare instances, an anomalous position of the kidney, or other organs, may be a source of fallacy: still, however, these deviations are not sufficient to interfere materially with our probable conjectures, though they must, of course, place a bar to that perfect certainty which it would be desirable to obtain, and which, at all events, it would be very satisfactory to look forward to, in such an important research."

Dr. Bright commences by pointing out with precision the boundaries of the various regions of the abdomen. The lines he draws differ a little from those of the best anatomical writers. Yet they seem to us unexceptionable, and rather better than those which occur at this moment to our recollection. The differences, however, between the boundaries of the regions which he proposes and those which are usually received, are too slight to induce us to quote his descriptions. When he speaks of the epigastric, or umbilical, pubic or lumbar regions, the reader may be satisfied with referring to the popular and familiar ideas of them. Nor need we quote his account of the organs and parts contained in each; the best works upon anatomy supply that information. Dr. Bright makes a very useful suggestion, which the majority of his and of our readers will, we fear, be more unanimous in admitting to be useful than in acting on. So prone to indolence are all, or, at least, so averse are all to adding to the fatigue of the ordinary duties and occupations of life.

"With a view of assisting in registering facts, it appears very desirable that every one, who is really anxious to make the most of the experience which comes within his reach—a duty which, unfortunately, from the time it occupies, we are all too apt to neglect—should provide himself with some ready mode of transferring to the corner or the blank page of his note-book an outline of the abdomen; upon which he may mark, as nearly as possible, the exact position of any tumor which he is called upon to treat: and, for this purpose, I have employed one or two different little contrivances, which it may not be amiss to mention. In the first place, having drawn on a thick sheet of paper the outline desired, we may, with a pin, make holes in a few prominent points; and pricking the note-book through these holes, the least-experienced draftsman will be enabled to make an intelligible sketch in a very short time. I have likewise had the figure cut in a brass plate, to use it in the mode of stencilling; and have thus procured, in a few seconds, upon any part of the page, such an outline as is represented in the figures in Plate II. Again, it would be a matter of a very few shillings' expense, to have a wood-cut or type formed, which might be used like a seal, even with common ink. It is obvious that no one single sketch can serve for every case; because the relative proportions of the different parts of the abdomen are somewhat altered, as it becomes distended, and consequently thrown out of its natural form; but still, the convenience of some such mechanical contrivance is very great, and there is no difficulty in being provided with more than one form of outline; and perhaps a second, representing the moderately-distended abdomen, would be quite sufficient for every purpose."

The first subject touched upon by Dr. Bright is that of:—

### I. ACEPHALOCYST HYDATIDS.

Dr. Bright, for convenience sake, arranges abdominal tumors resulting from hydatids, among the diseases of the peritoneum. Of course, this is not correct.

Whatever may be the origin of acephalocyst hydatids, they are most common somewhere in the region of the abdomen, and the liver, of individual organs, is, perhaps, the most frequently the seat of them.

Almost the only indication of their existence, continues our excellent friend, at the commencement, is the occurrence of swelling, corresponding with the part in which they are situated; and the gradual increase of this is, for a time, the chief mark of the progress which the disease is making. Occasionally, when a rounded projecting elastic tumor has been observed and felt for a time, a sudden subsidence takes place, accompanied by more or less constitutional and local excitement; and then the tumor may never arise again; or, instead of one definite tumor which had before been observed, several may appear to develop themselves within a limited period: at other times, the sudden disappearance of such a tumor may be followed by symptoms indicating peritoneal inflammation, so severe, as quickly to lead to dissolution. In a few instances, the subsidence of such a tumor is attended by the evacuation of hydatids, through the lungs, or the intestines, or in some other way, attended by various results.

The tumor, he continues, which presents itself externally, is most commonly, at first, distinctly referrible to the liver; and either occupies the right hypochondrium, or, protruding from beneath the ribs and their cartilages, encroaches upon the middle subdivision of the epigastric region, or descends into the right lumbar region, or approaches the umbilicus: its form is rounded, and its feel elastic; sometimes varying a little in the resistance it presents, being occasionally hard and even bony in some parts, and often indistinctly fluctuating on percussion. When situated in the right hypochondrium, it is sometimes accompanied by jaundice. When situated in other parts, the derangement of the functions of the particular organs upon which pressure is made will afford collateral indications not to be neglected. This form of disease is not confined to any age, or either sex; and the length of its continuance is not ascertained, though it is certain that it may exist for a great number of years without destroying life.

Neither situation, nor the sensation yielded to the touch, are sufficient to enable us to pronounce positively on the nature of hydatid tumors. It is necessary to weigh all the collateral circumstances, and, even then, the diagnosis is generally obscure, until the appearance in some way or some place of a hydatid sets all doubts at rest.

Dr. Bright relates, more or less circumstantially, fifteen cases of acephalocyst hydatids. Their nature may be gleaned from the following enumeration of them:—

Case 1. Hydatids in the Abdomen, of many years standing; shewing the acephalocysts in almost every stage of their existence.

Case 2. Hydatids extensively occupying the Abdomen.

Case 3. Hydatids developed in the Abdomen.—Death from Peritonitis.

Case 4. Tumor of the Abdomen, supposed to depend on the presence of Hydatids.

Case 5, Hydatid Cyst connected with the Liver. Death from suppuration of the sac and its consequences.

Case 6, Hydatid Cysts in the Liver ossified.

Case 7, Tumor in the Pubic Region from an Hydatid Cyst situated behind the Bladder.

Case 8, Tumor in the Pubic Region from an Hydatid Cyst situated behind the Bladder.

Case 9, Hydatid Cyst connected with the Liver, emptied by Paracentesis.

Case 10, Hydatid connected with the Liver emptied, by Paracentesis, with success.

Case 11, Hydatids in the Spleen bursting into the Abdomen, and causing death very speedily.

Case 12, Hydatid of the Liver, suppurating and bursting into the Abdomen, causing immediate death.

Case 13, Supposed Hydatid of the Liver bursting into the Cavity of the Abdomen.

Case 14, Hydatids in the Liver, supposed to have passed off by the Intestines.

Case 15, Hydatid Cysts in the Liver, discharging itself externally: and Death from Hæmorrhage.

We have not space, nor is it necessary, to present an account of each of these cases. We shall merely pick out such, or parts of such, as present remarkable features.

1. *Various States of Hydatids.* The first case is interesting, because on dissection it displayed a singular variety of the different states and conditions of the hydatid.

"The four principal masses into which these were distributed each afforded its remarkable peculiarity. In the large inferior tumor, the whole economy of the hydatid was in its most flourishing and healthy state. The parent, or protecting cyst, accurately lined the cavity in which it was contained; and the numerous progeny might be supposed to exult in the uninterrupted prosperity of their prolific community, and the pellucid medium by which they were surrounded. In all the other masses, or communities, some of the accidents to which the hydatid existence is subjected, were illustrated and proved.

In the small inferior cyst, placed behind the cyst of which I have just spoken, we found the parent hydatid separated from the cavity, that cavity contracted, and the hydatids crushed by the diminution of the cavity, and exposed to the influence of the absorbents of the body.

In the large superior cyst, the hydatids had apparently multiplied till they had perished for want of space: the parent cyst had died, the cavity suppurated, and the few hydatids which remained entire were floating in a mixture of pus and the decomposing *débris* of other hydatids: and here, likewise, the absorbents seemed to have been distended by the fluid of the cyst, and probably impeded in their action.

In the fourth cavity, the secretion of the liver had insinuated itself behind the parent cyst, destroyed its vitality, forced it from its situation, and, mingling with its contents, proved a source of death and destruction to the greater part of the hydatids it contained. Thus, as I have said, does this single case illustrate many of the most important epochs and accidents which mark the progress of the hydatid."

2. In Case 2, and Case 3, the hydatids would seem to have been, in the first instance, connected with the liver, and to have escaped from that viscus into the abdominal cavity. And yet, says Dr. Bright, it is observable, that none of these bodies seem to have effected a lodgment, or to have been developed, amongst the duplications of the mesentery and the convolutions of the intestines: and thus they have offered, comparatively, little interference to the peristaltic motions, and the general functions of the alimentary canal. It is probable that the immediate cause of this immunity is the close apposition of the convolutions, and the tendency which the intestinal motions may have to propel the little bodies forward; but in the pelvis, particularly behind the bladder, where a good opportunity of lodgment is afforded, we often meet with the hydatid cysts.

3. *Firm Adhesions of a Hydatid Cyst to the Abdominal Parietes—Death from Suppuration in the Cyst.* A woman, aged 42, had been twelve times jaundiced in twelve years. Six weeks before her admission into the hospital, she had another attack of jaundice, which went off in three weeks, and was succeeded by a rigor and low fever. There was a tumor at the pit of the stomach, extending almost to the umbilicus, tender on pressure, elastic to the feel, and appearing to have a solid base. This tumor, she said, had existed more or less for three or four years. Five days after her admission, she passed blood by stool, and in the course of twenty-four hours she died.

*Dissection.* The parietes of the abdomen were attached very firmly, by old adhesions, to the tumor, which occupied chiefly the right lobe of the liver: the tumor was of a membranous appearance, and vascular; and contained nearly a washhand-basin-full of hydatids, of all sizes, from that of a French walnut to a pea, but chiefly of about the size of a hazel-nut. The greater number were burst, and opake; but many retained their globular form. The fluid in which they were closely impacted was puriform; and the parietes of the large cavity were lined internally with a layer of thick pus-like matter, with shreds, and cakes of a cheesy substance, adhering closely. The cyst itself, owing to the suppuration that was going on, had assumed a worm-eaten appearance. The peritoneum displayed the usual marks of recent acute inflammation.

Two points of some consequence appear pretty certain in the preceding case:—*first*, that the patient died of suppuration in the sac and inflammation of the peritoneum; *secondly*, that the old-standing adhesions between the hydatid cyst and the abdominal parietes, would have rendered an operation, prior to the occurrence of suppuration in the sac, practicable, and might have made it successful.

4. *Ossification of Hydatid Cysts.*—A woman died with jaundice and mania. The liver was found to contain hydatid cysts; of which one, about the size of an orange, was completely ossified, throughout more than half its extent; and there were two or three smaller ones imbedded in the liver, which were not larger than a pea, but were also ossified; and all of them were filled with the remnants of hydatids, pressed together; and, in some parts, the convoluted laminæ were capable of being separated and unrolled.

5. *Tumor in the Pubic Region, from an Hydatid situated behind the Bladder.*—Dr. Bright mentions two cases of this sort. We shall quote one because it displays the symptoms which characterised and may characterise again this affection. It is therefore of practical value.

"The patient, who had been labouring under other disease, complained of the difficulty he had in retaining his water: and, when an examination was made, it appeared that the urine was continually passing away, and that a tumor, bearing all the characteristics of a distended bladder, presented itself at the pubic region. This at once suggested the idea that the patient was suffering from retention of urine, produced by enlarged prostate, or some such mechanical cause. A catheter was introduced by a skilful surgeon; and a few drams of perfectly healthy urine drawn off, without producing any diminution in the bulk of the tumor. As it was still supposed that urine was retained, more than one medical man attempted to draw it off; and at length the catheter became obstructed by the passing of small hydatids. When this was discovered, a sucking-pump was applied to the catheter, and thus a considerable quantity of the *débris* of hydatids was removed: however, the symptoms of this disease remained, his other complaints increased, and ultimately the patient died."

*Dissection.*—The tumor proved to be a large hydatid cyst, attached to the posterior part and the fundus of the bladder, and pressing so much forwards as to prevent entirely the bladder from being filled with urine; and this was the source at once of the tumor and of the constant escape of urine. The bladder itself was quite healthy; the catheter had passed by an opening from the urethra, behind the bladder into the cyst. This contained at least a quart of the shreds of burst hydatids. The tumor made pressure on the orifices of both the ureters, which were consequently very much distended with urine, and the pressure which had been made upon the kidneys by the fulness of the pelves had produced a very extensive absorption of the substance of both. The fluid contained in the ureters was puriform, and there were some small clots of blood in the infundibula.

Dr. Bright makes an observation which is very judicious. If, in drawing off the urine from what appears a full bladder, the former is not dark-coloured, nor of strong odour, that may at once induce us to suspect that the latter is not really distended.

6. *Hydatid connected with the Liver, successfully emptied by Paracentesis.*—A

young woman was admitted in December, 1828, with an elastic swelling occupying almost the whole scrobiculus cordis, and inclining rather to the left side; it was not the least discoloured, and gave an evident sense of fluctuation. The swelling was divided into two lobes, of which that to the left was the largest; and the fluid seemed to pass from one to the other. Two years before, an unavailing attempt had been made, to open it by caustic. Mr. Key now employed a small trochar, and four pints of a perfectly limpid fluid, with little or no smell, nor any appearance of lymph, shreds, or flocculent matter, were drawn off. The operation afforded her great ease, and was followed by no symptoms of inflammation. For a time, the cyst appeared to fill gradually; she lost her colour, and had frequent tendency to faint; and afterwards a little diarrhœa came on, which, however, was easily checked. After this, however, the tumor gradually diminished—the patient left the hospital at the end of February with only some fulness at the scrobiculus cordis—got married—and has had no re-accumulation of the fluid. From an analysis made by Dr. Bostock, it appears that 1000 parts of the fluid contained—

| | |
|---|---|
| "Water . . . . . . . . . . . | 987.5 |
| Extractive, with a trace of albumen . . . | 4 |
| Muriate of soda, with minute quantities of sulphuric acid and potash . . . . . . . . . | 8.5 |
| | 1000.0" |

After detailing the particulars of two cases, in one of which hydatids in the spleen, and in the other of which hydatid of the liver burst into the abdomen and proved immediately fatal, Dr. Bright details another instance of the same accident without the same disastrous result.

*Supposed Hydatid of the Liver bursting into the Cavity of the Abdomen.*—June 13, 1836. ——— ———, a boy of 14 years of age, was admitted into Barnabas Ward, under Dr. Addison, with the chest quite deformed by the protrusion of the lower ribs of the right side; but more particularly by the entire displacement of the false ribs, affording a sense of elasticity, and almost of fluctuation in that part. At the same time, the liver was pushed below his usual position, so that its margin was to be felt far down in the abdomen, below the umbilicus. It appeared most probable that this was an instance of hydatid cyst, situated on the convex surface of the liver.

At the end of two months he returned to his friends. In the latter part of August he suffered from hæmorrhage, after having a tooth drawn; and while recovering from this, in the beginning of October, he was attacked with severe pain in the swelling of the right side, and was very ill for several days; but recovered from that attack: and in the middle of the same month he applied as an out-patient at the hospital. Till the 25th of October the fluctuation remained the same, and the swelling increased a little; but on rising from his bed on that morning, he had a feeling of faintness, with a sudden sinking, and a peculiar motion, as he stated, in the tumor, which from that time greatly diminished in bulk, giving the conviction, to those who had seen him most, that the contents of the supposed hydatid tumor had escaped into the abdomen, without, however, producing any unpleasant symptom, except, perhaps, a little sickness at the stomach.

"Oct. 26. I saw him at his house: the tumor had subsided; but there was evident fulness and fluctuation at the pubic region and below the umbilicus. He had taken two or three doses of calomel, followed by sulphate of magnesia; and had passed a tolerably healthy loose dejection. He had experienced a little pain that afternoon in the lower part of the abdomen, but very little tenderness. His chief pain was in the act of passing urine, as if from the contraction of the bladder. Pulse about 140, sharp: tongue slightly furred: countenance flushed."

"28. I examined the abdomen very carefully, and found the margin of the liver much raised above its former position, descending only half-way to the umbilicus, and not apparently adhering to the diaphragm. The fluctuation at the lower part of the abdomen was diminished: there was neither tenderness nor pain. Bowels well open: he had scarcely moved from the same recumbent position, and had eaten nothing but a little gruel each day. Pulse 96. The ribs still bulged a little on the right side.

Rep. Pil. Hydrarg. gr. iij. Ext. Conii gr. iij. bis die."

After this the fluid decreased in quantity—he continued for some months in an emaciated state—but his state in Sept. 1837, was satisfactory:—"he is by no means thin, has some natural colour, the spine is straight, the abdomen not full, and the thorax is almost symmetrical.

He works for a printer; and has no complaint but that of a painful weakness in the left knee, which, as well as the right, seems to fall inwards, more than it has been wont to do.

The only other case we think it necessary to mention is the following. It seems to shew that hydatid cysts when they present at apertures, should not be much meddled with.

*Hydatid Cyst in the Liver, discharging itself externally, and Death from Hæmorrhage.*—Dr. Babington visited Mrs. Rastall, with Mr. May, of Bow Lane. Her skin was of a deep yellow hue; and her secretions sufficiently demonstrated an obstruction to the natural course of the bile. This state of jaundice was described to have existed for many months. On examining the abdomen, a hard tumor, irregular in figure, and rising into roundish masses, was felt on the right side, issuing from beneath the ribs, and extending to the pit of the stomach. There was no fluctuation in the tumor. Mrs. R. was pregnant. She gave birth to a healthy child, was again pregnant, went her full time, and bore a living infant. But the tumor progressively increased.

"In the beginning of December last, Mr. May again requested me to see Mrs. Rastall, and informed me, that a day or two before, the parietes of the abdomen had spontaneously given way, and an enormous discharge of fluid had issued from the aperture. On removing a poultice and bandage, which had been subsequently applied, I found an ulcerated opening, rather less than an inch in length, situated immediately below the umbilicus. From this opening a portion of what, at first view, bore a general resemblance to intestine, protruded about half-an-inch. On closer examination, its collapsed and puckered form, its total insensibility, and its greater thickness, led to a belief that it was the empty cyst of an hydatid; and, by gradual and gentle traction, I succeeded in bringing it away. The part which passed last was thinner, and more gelatinous in appearance, than the rest, and was torn in coming out. The whole was nearly as large as a bullock's bladder; and would probably have contained, previously to its rupture, a gallon and a half of fluid. No pain was felt during its abstraction; and the patient expressed herself as relieved by its removal. We saw her on the following day, and found that she had passed a good night. A second cyst now presented itself at the aperture; but as it seemed, on gentle traction, to be adherent, no force was used to withdraw it. The bandage with which the abdomen had been bound was soaked by a constant drainage of serous fluid. On the day following my second visit, blood, instead of serum, began to make its appearance; and this was accompanied by such a degree of prostration, rigor, and faintness, as led to the apprehension that some serious internal hæmorrhage was taking place. This was verified, as the day advanced, by more alarming seizures of syncope; and one of these proved fatal, in the course of the night. That hydatids had caused all the mischief in this case, was demonstrated during life; and an examination, after death, proved that one of these had formed in the interior of the liver, near its under surface; forcing, as it grew, the substance of that organ upwards and forwards, and its posterior peritoneal coat backwards. A large cavity, lined with a false membrane, and filled with grumous blood, was found thus bounded. A second hydatid, the cyst of which remained, seemed to have occupied a portion of the same cavity; and a third, about the size of a walnut, appeared on the convex surface of the right lobe."

Such are the more important of the cases detailed by Dr. Bright. The observations that he makes are judicious, discriminating, and instructive. Yet all that it seems necessary to extract from them relates to the subject of treatment. He remarks that:—

"While confined to one cyst, whether formed by a solitary hydatid, or by one which is productive, and therefore contains within it a number of others, I believe that an opening, or a puncture, offers the best chance of cure: and if the cyst be solitary, it is not unlikely that the result will be satisfactory. If, on the contrary, the cyst contain a great number, much risk will be incurred, lest some of the excessively minute bodies should find their way into the peritoneal cavity; where, in all probability, though they might not produce any intense inflammatory action, they would gradually develop themselves and multiply. On the other hand, there is great danger in the existence of such a cyst, lest it should burst suddenly into the abdomen; and then the extent of mischief is incalculable: so that, supposing our diagnosis quite certain, there would be the greatest justification in performing the operation. As, however, our diagnosis in a single cyst is infinitely more difficult than when the disease becomes diffused, it will be always right to employ an exploring needle, such as has been recommended by Dr. Davies in cases of empyema, before an opening is made. Should the operation be performed, the most careful treatment must afterwards be adopted, to prevent any portion of the fluid, which will almost certainly be left behind in the cyst, from passing into the abdomen.

In what way the puncturing of an hydatid cyst might be expected to prove useful, is a question worth inquiry. If the cyst contain numerous hydatids, it will be no easy matter to make such an opening as would allow the larger of these bodies to escape, without too great a risk to the patient's life; but perhaps this might not be necessary to the success of the operation. I imagine one of the best results which could arise from the operation, would be the destruction of the parent or protecting cyst. Whether a simple puncture would effect that purpose, I cannot say; but probably it would; more especially as, when a considerable portion of the fluid contents of the cavity was withdrawn, the cyst would be likely to fall in, and separate from the parietes of the cavity, and thus subject the whole contents to the influence of absorption: but this result would be more certainly obtained, if a more extensive rent or separation could be inflicted on the hydatid. In the cases which have been detailed, there are three instances of the external discharge of the hydatid, besides those discharges which have taken place by the intestines and the lungs. Of these cases, one was spontaneous, and was followed by hæmorrhage from the cyst after the separation of the hydatid. In another case suppuration was far advanced in the cyst before recourse was had to the operation. In the third the operation was performed upon an hydatid which proved apparently to be solitary and unproductive, and a cure was effected. Here, probably, the cyst was destroyed; and being separated from the walls of the cavity, which were healthy, the absorbents acted as far as was necessary, while the cavity contracted.

The chief good which it appears likely that medicine can effect, is to excite the action of the absorbents when the parent hydatid is dead or separated: for till that preliminary step is obtained, the absorbents may act upon the system, but are not likely to act upon the hydatid, or the fluid contained within it; and it would be a legitimate object in the administration of medicines, to destroy the life of the parent hydatid: but, as yet, we know little upon this point of treatment."

Dr. Bright suggests, but does no more than suggest, repeated doses of turpentine, electricity, the application of ice. The whole paper is a valuable one.

### II. Ovarian Tumors.

This paper, occupying 89 pages, is contained in a succeeding, the sixth Part, of the Reports.

1. Dr. Bright observes that there are, perhaps, four distinct diseases which form pelvic tumors with fluid contents, and which are therefore spoken of as ovarian dropsies. The first presents itself as a simple bag, containing serum; whose external surface appears to possess all the attributes of the peritoneum, attached to the surface of the ovary or some neighbouring part, and supplied by blood-vessels from the point whence it arises; sometimes, sessile; more frequently attached by a longer or shorter neck; generally single; but occasionally presenting the appearance of being composed of more than one cyst. This simple cyst, with a long footstalk, is not of unfrequent occurrence; and is sometimes congenital, or at least exists within a few months after birth. The tumor is generally of small dimensions, from a size less than a pea, to that of an orange; probably, at times, to a larger. The sessile variety, continues Dr. B., of the simple cyst often develops itself into the broad ligament; and appears still more decidedly placed beneath the natural fold of peritoneum than the last variety, so as apparently to involve within it the Fallopian tube; which, however, is found passing round it, and not materially altered from its natural state, or slightly dilated, but not communicating with the cyst.

2. "The second source of tumor containing fluid, connected with the uterine appendages, is found in a distended state of the Fallopian tube, which is not unfrequently seen obstructed and filled with serous fluid, so as to be much dilated, forming a pouch of considerable size; and often both of the Fallopian tubes are similarly affected. Whether, however, the dilatation is ever of such dimensions as to present a very distinct elevation above the pubes, I cannot say from my own observation: I have much more frequently found these sacs capable of containing a few drams, or at most an ounce or two, of fluid. One, which is preserved in the Museum of the College of Physicians, and of which a plate has been published in Dr. Seymour's excellent treatise on the diseases of the ovary, is five or six inches in length, and would probably contain half-a-pint of fluid."

Dr. Bright alludes, en passant, to the distended state of the Fallopian tube, which sometimes occurs from purulent or scrofulous deposits. But he has never seen this of sufficient size to form a decided abdominal tumor.

3. "The third form of tumor, the existence of which, as a separate disease distinct from others, appears to me very doubtful, consists of a simple vesicular body, developed beneath the proper tunic of the ovary; supposed to be produced by an accumulation of fluid in one of the Graafian vesicles. Tumors, said to be of this kind, differ greatly in size: they are frequently not larger than a hazelnut; sometimes of the size of an egg; and occasionally are believed to attain to a great magnitude, so as nearly to fill the abdomen. Yet, from the description which has sometimes been given of the contents of such supposed cysts and their glutinous quality, it is probable that they have often been no other than largely developed cysts of a malignant character. There is a state of the Graafian vesicle by no means uncommon; and of which we have several specimens in the Museum of Guy's, where a coagulum, more or less stained with blood, or of a somewhat glutinous character, is collected in the vesicle, distending it to the size of a hazel-nut, or sometimes larger : this, likewise bears a doubtful relationship to the malignant forms of tumor ; but from the circumstances of some of the patients and their youthful age, I suspect that they are, at least occasionally, unconnected with such disease."

4. The fourth, proceeds our able author, is by far the most frequent form of ovarian tumor; and is essentially a specific disease, assuming all the varieties of structure which result from the numerous modifications of that morbid action called malignant. It is in the cellular tissue of the ovary that this malignant disease originates, a fact on which Dr. Bright insists ; yet he owns that it is often quite impossible to say whether it be the meshes of the cellular tissue, or the vesicles of De Graaf, which are become the seats of the morbid deposits, or to what extent new structures have been generated.

Dr. Bright dwells (how could he do otherwise?) on the propriety, the advantage of discriminating, if possible, between the symptoms of a simple cyst, and those of malignant disease of the ovary. But he admits that that discrimination is difficult.

"I do not profess," he says, "to be conversant with the history of the simple cyst: I believe that the only indication afforded is a tumor, mose or less spherical, felt first in one of the inguinal regions of the abdomen, and very gradually, if at all, ascending. That this may take place very early in life—that its growth is slow—that the constitution suffers little or not at all—and that after the cyst has attained even a large size, it occasionally disappears, without any very evident cause, or under the action of remedies, or from being burst internally by some accidental occurrence—the fluid, in this last case, passing off by the Fallopian tubes, or taken up by the absorbents, and hurried very rapidly through the kidneys ; and though this effusion of fluid may be accompanied by a certain degree of peritonitis, the symptoms are, in general, by no means so alarming as might be expected. I am not sure that I can recall to my memory a single dissection where the simple ovarian cyst has been the cause of death, or has even advanced to such a size as to be the subject of material inconvenience to the patient during life. In most of the cases—and they are pretty numerous—in which simple cysts have been discovered after death, they have been too small to have attracted notice during life, and have been casually detected. Their attachment has been even more frequently to the Fallopian tubes, or to their fimbriated extremity, than to the ovaries themselves; and they have seldom exceeded the size of a small plum. The subjects in which they have occurred have varied, as to age, from children in arms, to women in the decline of life ; and in these latter cases, though still small, they have often borne, in their structure, such evidence of having long existed, that I am inclined to believe their increase to be generally very slow ; and that they frequently become stationary at an early period.

With regard to the accumulation of fluid in a Graafian vesicle, leading to its gradual distention, this likewise cannot easily be detected ; and, if detected, must with great difficulty be distinguished from other forms of ovarian tumor. As soon as it has acquired a sufficient size, it will, of course, be felt rising from the pelvis ; less spherical, and less moveable, than the simple cyst ; less lobulated than the malignant disease : which circumstance, together with its more moderate growth, and the little inconvenience it produces, may afford a clue to our diagnosis, and guard us against an inordinate anxiety for the result. It is probable, however, that this form of tumor more frequently attains a large size that the simple cyst ; and that it more frequently affords those instances of sudden disappearance, by accidental rupture of the cyst, or of gradual decrease, assisted by medicine, than any other form of ovarian growth. When rupture takes place, there is very often a more or less acute inflammatory action induced ; and, according to the various circumstances arising from that inflammation, the succeeding history of the disease will be modified. Still, it is a fact, that from a very early period of its history, to the very end, we are not only unable to make any decided distinction between it and the malignant disease during life; but are seldom able to demonstrate, even after death, the precise nature of the tissue in which the cyst has been first developed."

These are candid admissions and judicious statements. The experience of most men will bear out Dr. Bright. He next passes to the more ordinary form of ovarian tumor, and presents a brief account of its prominent points.

It seldom shews itself much before the twentieth year of life. The first recognized symptom is usually a tumor, not altogether devoid of pain, in one of the inguinal regions; and which, on examination, evidently rises out of one side of the pelvis, and even at this early period is sometimes distinctly lobulated or uneven in its form, and unequal in the resistance its different parts afford on pressure. The growth of this tumor is, on some occasions, so unperceived, that though it may have originated on one side, it has already risen into the pubic, and even the umbilical region; and when the medical man is first consulted, its lateral origin is with difficulty ascertained. At other times, the enlargement is at first slow; and after some indefinite period, the increase takes place suddenly; so that, in a few months, the whole abdomen presents to a common observer, the size and appearance of pregnancy far advanced. After this the tumor may seem not to advance, but it grows more tense, the respiration is more embarrassed, the lower extremities grow anasarcous, and the operation of paracentesis is obviously necessary. Dr. Bright gives the following hints on the mode of examining and determining the nature of the abdominal tumor.

" Casting the eye over the abdomen in the earlier part of the disease, the greater rotundity or projection of one part will often be most apparent; and the tumor will in this way immediately discover itself, as occupying the iliac and lumbar region on one side, and extending over half at least of the umbilical region, or beyond the umbilicus, so as to encroach on the opposite side: at other times, its extent will be less; while in the more advanced cases no inequality will strike the eye; but the rounded form of the abdomen, while the patient lies on her back, will contrast it with the more ordinary ovoid appearance of ascites, as well as distinguish it in some degree from the form produced by the uterus distended in pregnancy. In many cases moreover, the eye will be struck by a great enlargement of the subcutaneous veins, as I have just observed, and such as often takes place to a still greater degree in ascites.

On making more firm though not violent pressure on the various parts of the abdomen, we often find at once the general sense of fluctuation; and ascertain inequalities which neither the eye, nor the hand when passed but gently over the surface, will enable us to detect: and then it sometimes happens, particularly if the abdomen be not very tense, that we discover considerable masses of unyielding matter, partaking of the general rounded feel of the whole disease, but conveying the impression of more or less flattened spherical bodies attached to the inside of the fluctuating tumor; and these bodies are sometimes so large, and sometimes so variously placed, as to suggest, to the inexperienced observer, the idea that the liver, the spleen, or the kidneys, are enlarged, and in some way involved in the disease."

" Sometimes the sense of fluctuation is very indistinct or very partial, and various parts of the tumor yield it in different degrees. At other times, the fluctuation is even more evident than in the most extreme cases of ascites; and sometimes, as the patient lies on the back, a thin layer of fluid is discoverable, external to the great distending tumor; so that when the points of the fingers, placed on the surface, are moved forwards with a jerk, they are evidently resisted, after pressing aside a little fluid, by the surface of the tumor within. Sometimes this layer of fluid extends over a wide space; and the fluctuation it yields by percussion may be plainly felt, or even seen as a wave passing over a large portion of the abdomen."

Percussion is valuable. In ascites, the intestines usually float upon the surface of the fluid, and are distinguished by the tympanitic sound at the highest part, whatever the position of the patient. But, in ovarian disease, the viscera are displaced, and retain their position under all circumstances.

The state of the subcutaneous cellular membrane should be investigated, to ascertain whether there are any tubercular deposits in it. The existence, too, of adhesions between the tumor and the peritoneum should be a matter of inquiry. To all this should be added an examination par vaginam.

The fluid drawn off by operation is glairy, or mucilaginous, or dark, or turbid, or loaded with cholesterine. When the fluid is nearly withdrawn, floating as it were, in the flaccid abdomen, one or perhaps two or three hard bodies are discovered; one round; another flat, and shaped like a placenta. The fluid now accumulates still more rapidly. In a few months it must again be drawn off. Its character is often changed: it is more gelatinous or more opaque, sometimes becoming puriform; or it is mingled with blood or cerebriform matter. Operation succeeds to operation, with diminished intervals; the constitution sympathizes; and, after a limited number of months or years, the patient sinks, exhausted by weakness, overcome by inflammation, or worn out by pain.

Our Author relates, more or less fully, the particulars of twenty-six cases. Their number precludes an account of them, but a notion of their nature and of the facts which they exhibit may be gathered from an enumeration of their titles. The first two cases are instances of simple cyst, in one growing from the appendages of the uterus on the left side, in the other attached to the ligaments of the uterus on each side.—Case 3, is Incipient Ovarian Dropsy, probably of a malignant character.—Case 4, Diseased Ovary, with Cysts, in a case of extensive malignant disease of other organs.—Case 5, Ovarian Dropsy of eleven years' standing.—Death probably from inflammatory changes going on in the interior of the cyst.—Case 6, Ovarian Dropsy, Anasarca, and Ascites.—Death from Peritonitis, after the fluid was partially drawn from the cyst.—Case 7, Ovarian Dropsy of many years' duration; shewing several cysts in different conditions;—with the analyses of the fluids they contained.—Case 8, Malignant Tumor in Abdomen, probably Ovarian—discharging constantly from the wound made by paracentesis.—Case 9, Compound Ovarian Cyst—death from exhaustion.—Case 10, Compound Ovarian Cyst—the fluid never removed—slow exhaustion, and death—adhesions of the tumor to the parietes discerned during life.—Case 11, Compound Ovarian Cyst—the fluid never removed—death from irritation and exhaustion.—Case 12, Ovarian Dropsy, fatal from Peritonitis after the partial abstraction of the fluid.—Case 13, Cyst, probably a diseased Graafian vesicle, communicating by ulceration with the colon—death from irritation of the mucous membrane of the large intestine.—Case 14, Ovarian Cyst, probably a diseased Graafian vesicle, fluid partially removed—inflammation of cyst and peritoneum.—Case 15, Ovarian Dropsy—paracentesis—death from peritoneal inflammation.—Case 16, Cyst in the broad ligament of the Uterus—ruptured—death sudden.—Case 17, Ovarian Tumor, partly fluid, partly fungoid—paracentesis rendered necessary, from the great pain experienced—death, after several operations, from peritoneal inflammation.—Case 18, Ovarian Dropsy—possibly enlarged Graafian vesicle—rupture of the cyst internally—death from inflammation of the cyst and neighbouring parts—the cyst tympanitic.—Case 19, Malignant Ovarian Cyst ruptured internally—subsidence of the tumor—death in about two years, from increase of the disease.—Case 20, Ovarian Cyst ruptured internally—death after three years, with emaciation.—Case 21, Compound Ovarian Cyst, ruptured internally; followed by death from peritonitis.—Case 22, Malignant Disease of the Ovary—fibrous tubera in the uterus—paracentesis—subcutaneous tubera on the abdomen—death from extensive scirrhous disease.—

Case 23, Ovaries affected with a modification of the malignant disease.—The peritoneum extensively involved in similar affection.—Case 24, Ovarian Tumor, with extensive growth of pendulous malignant tumors from the peritoneum.—Case 25, Malignant Ovarian Tumor, with Ascites, communicating disease by contiguity to the Sigmoid Flexure and Rectum.—Case 26, Ovarian Dropsy.—Cerebriform disease, communicating to contiguous organs.—Case 27, Hysterical distention of the bowels, mistaken for Ovarian tumor—Operation to attempt its removal.

Appended to the Cases are some general observations on the diagnosis, prognosis, and cure of ovarian tumors. The following hints on the subject of diagnosis may be quoted.

"The circumscribed extent of the tumor, and consequently of the fluctuation, distinguishes all ovarian cysts, in the early stages, from ascites. The fluctuation, uninterrupted by the intestines in any part, distinguishes the disease in more advanced cases. The lateral situation of the tumor, in the early stages, distinguishes it from pregnancy in the normal state of the viscera. Its duration distinguishes it in the more advanced stages: the suppression of the catamenial discharge adds probability to the existence of pregnancy; but there is no certainty to be derived from this indication; as in ovarian disease the catamenia are sometimes regular, sometimes irregular, sometimes wanting: alterations in the mammæ are likewise uncertain indications; and in doubtful cases, nothing, except examination by the vagina, can give tolerable certainty; and then the shortened cervix, and the weighty feel of the uterus, would decide the question in favour of that organ. From the malignant disease of the fundus of the uterus, the situation will in part distinguish it: the hardness of the tumor, and the peculiar abrupt nodules which the diseased uterus presents, contrast well with the soft and yielding feel which the subsidiary tumors of the compound ovarian cyst usually afford. The origin of these cysts from the pelvis generally distinguishes them from all tumors of the abdomen, except diseases of the uterus, or a thickened or distended bladder; and the central situation of both these viscera suffices, for the most part, to fix disease upon them, when it exists."

In reference to Prognosis, it is impossible to abridge this already abridged passage:—

"We find, in the cases above recorded, that the malignant ovarian tumor, left to itself, often destroys life in a short time, partly by irritation, but probably in a great degree by the mechanical pressure it exerts (Cases 10 and 11). That if paracentesis is performed, life may be prolonged; but that sometimes, from the inflammation excited by the circumstances attending the operation (Cases 6, 12, 14, 15, 17, &c.), and sometimes without any discernible inflammation (Case 9), death takes place. That in other cases, the malignant disease seems to undermine the constitution, and gradually leads to a fatal result. (Cases 23, 24, 25, 26). That, occasionally, the internal rupture of the cyst produces death. (Cases 16, 18, 19.) But still, with such a discouraging prognosis before us, we have every reason to feel that the interposition of our remedial means is productive of great alleviation, and is capable of prolonging life; and although the duration of this disease is often limited to months, and still more frequently to a very few years, yet instances are not wanting, in which ten, twelve, or a still greater number of years have been passed in tolerable comfort: and in giving our prognosis, we should suffer ourselves and our patients to have the full benefit of the hope which such cases are calculated to inspire."

On the treatment and the expectation of cure, Dr. Bright does not, nor can he say much. He alludes to the operation of excising the tumor as a doubtful and dark operation. And so it is. It may succeed, but a common operation it never can be. Dr. Bright recommends, of course, attention to the secretions, the mitigation of pain, the setting right, as far as possible, of what is evidently wrong by the usual and appropriate means.

Iodine, he thinks, should be given with care, and much should not be expected from it.

Of paracentesis Dr. Bright speaks at large. An early operation is not recommended by him, and for obvious reasons.

"I conceive," he says, "that the time for the operation is arrived when the tumor pretty fairly occupies a large portion of the abdomen, giving the appearance of pregnancy advanced to the last months, and before any material mischief seems to threaten, either to the surrounding viscera or to the parietes of the tumor itself: for there can be little doubt that the forcible distention of the sac, continued beyond a certain limit, will endanger its inner surface, and, perhaps, prove one cause for those ulcerative changes which often take place and are the source of great constitutional irritation, and of death, as seen in several of the foregoing dissections."

The part at which the puncture is to be made is a matter worthy of consideration.

The part in which the fluid is chiefly accumulated must, as far as possible, be ascertained. For Dr. B. has known instances in which the operator has been foiled, and even obliged to make a second puncture, from having pushed the trochar into a solid mass: and even when distinct fluctuation has been ascertained, the thickness of the fluid has sometimes prevented its passing by the puncture; or, by opening into some secondary cyst, a few ounces only have been drawn off. In one of Dr. Bright's cases, another source of disappointment resulted from the trochar passing into a firm band formed by the broad ligament and the Fallopian tube stretched over the tumor; and affording such resistance, that the instrument pushed the parietes of the tumor before it; and when withdrawn, under the belief that it had gone deep into the cyst, no fluid escaped.

It is right, continues our author, before operating, to be quite sure that the bladder is empty; and, of course, enlarged veins must be carefully avoided: and, in general, it will be right to examine the state of the uterus per vaginam; more particularly if it be the first time that the operation has been performed. Should the sensation given by pressure on the abdomen lead to the belief that adhesions have taken place between any portion of the cyst and the parietes, it will be well to select this part for the operation, as we shall thus avoid one important source of danger.

With the following directions we conclude:—

"Another very important point, is the quantity of fluid which should be drawn away. Some there are who think that it is most advisable to take away but a small portion of the whole, just sufficient to relieve the urgent oppression; supposing that by this means the cyst is more likely to contract, and believing that the fluid does not re-accumulate so rapidly in this case as where the distending contents are at once withdrawn. On the whole, however, I generally recommend that as much of the fluid as possible should be removed; because I dread, above every other danger, that of a portion of the fluid escaping into the peritoneum; of which we run a great risk, if the wounded parietes of the tumor fall in upon a considerable quantity of fluid: and in order to avoid this still further, a regular gentle pressure should be maintained, as is usual during and after the operation in ascites; and for some days the most perfect tranquillity should be enjoined, the patient being treated like one who is recovering from labour."

We hope Dr. Bright will continue to furnish such papers as these to the profession.

